# Association of the c.385C>A (p.Pro129Thr) polymorphism of the fatty acid amide hydrolase gene with anorexia nervosa in the Japanese population

**DOI:** 10.1002/mgg3.69

**Published:** 2014-02-24

**Authors:** Tetsuya Ando, Naho Tamura, Takashi Mera, Chihiro Morita, Michiko Takei, Chiemi Nakamoto, Masanori Koide, Mari Hotta, Tetsuro Naruo, Keisuke Kawai, Toshihiro Nakahara, Chikara Yamaguchi, Toshihiko Nagata, Kazuyoshi Ookuma, Yuri Okamoto, Takao Yamanaka, Nobuo Kiriike, Yuhei Ichimaru, Toshio Ishikawa, Gen Komaki, The Japanese Genetic Research Group For Eating Disorders

**Affiliations:** 1 Department of Psychosomatic Research, National Institute of Mental Health, National Center of Neurology and PsychiatryKodaira, Tokyo, Japan; 2 Department of Psychosomatic Medicine, Kohnodai Hospital, National Center for Global Health and MedicineIchikawa, Chiba, Japan; 3 Division of Psychosomatic Medicine, Department of Neurology, University of Occupational and Environmental HealthKitakyushu, Fukuoka, Japan; 4 Department of Psychosomatic Medicine, Yahata Kosei HospitalKitakyushu, Fukuoka, Japan; 5 Department of Psychosomatic Medicine, Graduate School of Medical Sciences, Kyushu UniversityFukuoka, Fukuoka, Japan; 6 Takei Medical ClinicKagoshima, Kagoshima, Japan; 7 Department of Psychosomatic Medicine, Saitama Social Insurance HospitalSaitama, Saitama, Japan; 8 Department of Psychosomatic Medicine, Kamibayashi Memorial HospitalIchinomiya, Aichi, Japan; 9 Health Services Center, National Graduate Institute for Policy StudiesMinato-ku, Tokyo, Japan; 10 Department of Psychosomatic Medicine, Nogami HospitalKagoshima, Kagoshima, Japan; 11 Department of Psychosomatic Medicine, Family Hospital SatsumaSatsumasendai, Kagoshima, Japan; 12 Division of General Medicine, Aichi Medical University HospitalNagakute, Aichi, Japan; 13 Setoguchi Psychosomatic ClinicSeto, Aichi, Japan; 14 Department of Neuropsychiatry, Osaka City University Graduate School of MedicineOsaka, Osaka, Japan; 15 Mental Health Clinic of Dr. Nagata at NanbaOsaka, Osaka, Japan; 16 Department of Internal Medicine, Yufuin Koseinenkin HospitalYufuin, Oita, Japan; 17 Health Service Center, Hiroshima UniversityHigashihiroshima, Hiroshima, Japan; 18 Graduate School of Welfare Society, The International University of KagoshimaKagoshima, Kagoshima, Japan; 19 Nishihara HoyouinKaya, Kagoshima, Japan; 20 Hamadera HospitalTakaishi, Osaka, Japan; 21 Department of Nutrition, School of Home Economics and Science, Tokyo Kasei UniversityItabashi-ku, Tokyo, Japan; 22 School of Health Sciences at Fukuoka, International University of Health and WelfareOhkawa, Japan

**Keywords:** Anandamide, cannabinoid 1 receptor, eating disorder, endocannabinoid

## Abstract

The functional c.385C>A single-nucleotide polymorphism (SNP) in the fatty acid amide hydrolase (FAAH) gene, one of the major degrading enzymes of endocannabinoids, is reportedly associated with anorexia nervosa (AN). We genotyped the c.385C>A SNP (rs324420) in 762 lifetime AN and 605 control participants in Japan. There were significant differences in the genotype and allele frequencies of c.385C>A between the AN and control groups. The minor 385A allele was less frequent in the AN participants than in the controls (allele-wise, odds ratio = 0.799, 95% confidence interval [CI] 0.653–0.976, *P* = 0.028). When the cases were subdivided into lifetime restricting subtype AN and AN with a history of binge eating or purging, only the restricting AN group exhibited a significant association (allele-wise, odds ratio = 0.717, 95% CI 0.557–0.922, *P* = 0.0094). Our results suggest that having the minor 385A allele of the *FAAH* gene may be protective against AN, especially restricting AN. This finding supports the possible role of the endocannabinoid system in susceptibility to AN.

## Introduction

The endocannabinoid system is comprised of the cannabinoid 1 and 2 receptors (CB1R and CB2R, respectively), endogenous ligands (e.g., N-arachidonoyl-ethanolamide [AEA], also known as anandamide, and 2-arachidonoyl glycerol [2-AG]), and enzymes for ligand biosynthesis and inactivation (e.g., fatty acid amide hydrolase [FAAH], N-acylethanolamine acid amidase [NAAA], and monoacylglycerol lipase [MGL]) (di Marzo 2008). The endocannabinoid system modulates neurotransmission at synapses and is involved in the regulation of many central and peripheral functions, including appetite, food intake, and energy balance (Pagotto et al. 2006). Dysregulation of the endocannabinoid system has been implicated in the neurobiological basis of neuropsychiatric disorders, such as anxiety disorders, depression, schizophrenia, and eating disorders (Marco et al. 2011).

Changes of the endocannabinoid system in eating disorders have been suggested. Increased blood levels of AEA were found in both anorexia nervosa (AN) and binge-eating disorder patients, but not in bulimia nervosa (BN) patients, whereas no significant change occurred in the plasma levels of 2-AG (Monteleone et al. 2005). Elevated levels of CB1R but not CB2R mRNA were found in the blood of females with AN and BN (Frieling et al. 2009). In addition, the availability of CB1R in the brain was reportedly altered in AN and BN patients (Gerard et al. 2011).

FAAH is a membrane enzyme that inhibits the activity of fatty acid amides, including AEA, which is the main ligand for CB1R (McKinney and Cravatt 2005). CB1R mediates many actions of AEA such as its effects on appetite, food intake (Berry and Mechoulam 2002), emotion (Martin et al. 2002; Kathuria et al. 2003), and pain (Lichtman et al. 2004). Disruption of the *FAAH* gene dramatically elevates the endogenous levels of fatty acid amides in the central nervous system, resulting in CB1R-dependent behavioral responses (Cravatt et al. 2001) and the promotion of energy storage and hypoalgesia in mice (Lichtman et al. 2004). Similarly, the administration of FAAH inhibitors, which causes a significant increase in the brain levels of fatty acid amides, exerts CB1R-mediated anxiolytic effects in rodents (Kathuria et al. 2003).

The natural missense c.385C>A single-nucleotide polymorphism (SNP) (rs324420) in the human *FAAH* gene (OMIM: 602935) substitutes a conserved proline residue at amino acid position 129 to threonine (Sipe et al. 2002). It was shown that the mutant FAAH enzyme has ∼50% the cellular expression and activity of the wild-type protein due to the reduced stability of the mutant protein (Chiang et al. 2004). This may result in the reduced inactivation of AEA, increased AEA levels in the central nervous system, and eventually enhanced CB1R-mediated actions.

It was reported that the *FAAH* c.385C>A SNP had an influence on weight loss and insulin resistance after the administration of a high monounsaturated fat hypocaloric diet (de Luis et al. 2013). In eating disorders, Monteleone et al. (2009) showed that the *CNR1* (the gene for CB1R; OMIM: 114610) c.1359G>A and *FAAH* c.385C>A SNPs were significantly associated with AN and BN, and that there was a synergistic effect of both SNPs on AN. However, Müller et al. (2008) did not find such an association of these SNPs with AN in their transmission/disequilibrium test (TDT) and case–control study.

The purpose of this study was to examine if the functional *FAAH* c.385C>A SNP is associated with AN in a Japanese population using a case–control approach.

## Methods

The participants were 763 unrelated Japanese female patients with a reliable history of AN clinically diagnosed by a psychosomatic physician or psychiatrist who was experienced with eating disorders. Diagnosis was made according to the criteria of the Diagnostic and Statistical Manual of Mental Disorders (DSM-IV), but amenorrhea was not required. All patients were recruited from facilities collaborating in the Japanese Genetic Research Group for Eating Disorders. Their mean age, mean current body mass index (BMI), and minimum BMI were 25.1 ± 8.5 (SD) years, 16.0 ± 3.1 kg/m^2^, and 12.8 ± 2.1 kg/m^2^, respectively. According to the DSM-IV criteria, 370 subjects were currently diagnosed as AN restricting type, 235 as AN binge-eating/purging type, 60 as BN purging type, 17 as BN nonpurging type, and 32 as eating disorder not otherwise specified. Forty-nine subjects were in remission. Unrelated Japanese female volunteers were recruited among university students as control subjects after excluding those who reported a history of an eating disorder, any other psychiatric illness, or a metabolic, endocrine, or gastrointestinal illness that could affect body weight, resulting in data from 606 nonclinical control subjects. Their mean age was 20.8 ± 2.0 (SD) years, mean current BMI was 20.5 ± 2.3 kg/m^2^, and lifetime minimum BMI was 19.3 ± 1.9 kg/m^2^.

The ethics committee of the National Center of Neurology and Psychiatry, as well as those at all other collaborating facilities, approved this investigation. All subjects gave their written informed consent prior to participation in the study. Parental consent was obtained for minors.

The lifetime diagnosis and subtyping of AN were made according to the criteria of Pinheiro et al. (2010), in which amenorrhea is not required for any AN diagnosis, with slight modifications. The definitions of lifetime diagnoses used in this study are shown in Table 1. Using the criteria, the 376 patients were categorized as AN restricting subtype (RAN), 42 were AN purging subtype (PAN), 210 were AN with binge eating (BAN), 135 were lifetime AN and BN (ANBN).

**Table 1:** Abbreviations and definitions of the AN groups used in this study

Abbreviation	*N*	Name	Description
RAN	376	Lifetime anorexia nervosa restricting subtype	Lifetime history of DSM-IV^1^ AN-R subtype only. No history of AN-BP or BN.
PAN	42	Lifetime anorexia nervosa purging subtype	Lifetime history of DSM-IV^1^ AN-BP subtype with purging behavior, without binge eating. No history of BN.
BAN	210	Lifetime anorexia nervosa with binge eating	Lifetime history of DSM-IV^1^ AN-BP subtype with binge eating, with or without any purging behavior. No history of BN.
ANBN	135	Lifetime anorexia nervosa and bulimia nervosa	Lifetime history of any DSM-IV^1^ AN subtype and, at different time, BN.

AN, anorexia nervosa; BN, bulimia nervosa; BP, binge eating or purging; DSM-IV, Diagnostic and Statistical Manual of Mental Disorders; R, restricting.

1 Amenorrhea not required for any diagnosis of anorexia nervosa.

Peripheral whole venous blood was collected in a heparinized tube and stored for genetic analysis. Clinical records, including the information necessary for diagnosis and subtyping, were obtained, and data on current age, height, and weight, and past minimum weight at adult height were collected.

Genomic DNA was extracted from peripheral blood using a standard procedure. Genotyping of the c.385C>A (p.Pro129Thr) SNP (rs324420) in *FAAH* (OMIM: 602935, the reference sequence accession number: AL122001.32) was performed using TaqMan SNP Genotyping Assays with the ABI PRISM 7900HT Sequence Detection System (Applied Biosystems, Foster City, CA) according to the manufacturer's instructions.

The chi-squared test was used to determine whether the observed genotype frequencies deviated from the Hardy–Weinberg equilibrium (HWE) in the case and control samples. Comparisons of genotype and allele frequencies between groups were also performed using the chi-squared test. Comparison of BMIs was done with one-way analysis of variance (ANOVA). SAS System for Windows 9.3 (SAS Institute Japan Co., Tokyo, Japan) was used. The statistical power of our sample size was calculated using power calculator for Genome-Wide Association Studies (GWAS; Skol et al. 2006). Because the C to A substitution at *FAAH* c.385C>A was shown to reduce FAAH activity and because disruption of FAAH function has been implicated in an elevation of AEA in the brain, resulting in increased food intake and decreased anxiety, we hypothesized that the A allele of the c.385C>A SNP of *FAAH* would be less frequent in AN patients.

## Results

A total of 1369 participants were assayed and 1367 (call rate 99.9%) were genotyped successfully, resulting in 762 cases and 605 controls. The distribution of the *FAAH* c.385C>A genotypes did not deviate from HWE in the healthy controls (*χ*^2^ = 0.31, *P* = 0.86) or AN patients (*χ*^2^ = 0.58, *P* = 0.75).

There were significant differences in the genotype and allele frequencies of the c.385C>A SNP between all of the AN patients and the control group (genome-wise, *χ*^2^ = 6.49, *P =* 0.039, allele-wise, *χ*^2^ = 4.82, *P* = 0.028, odds ratio = 0.799 [0.653–0.976]). The odds ratio of a 385A allele carrier for AN was 0.747 (0.593–0.941) (*χ*^2^ = 6.16, *P* = 0.0131). When the AN patients were subdivided into lifetime RAN (*n* = 375) and other lifetime diagnoses (PAN, BAN, and ANBN) (*n* = 387), a significant association was observed only between the RAN patients and the control group (genome-wise, *χ*^2^ = 7.28, *P* = 0.026, allele-wise, *χ*^2^ = 6.75, *P* = 0.0094, odds ratio = 0.717 [0.557–0.922]). The odds ratio of a 385A allele carrier for RAN was 0.676 (0.508–0.899) (*χ*^2^ = 7.26, *P* = 0.0071) (Table 2).

**Table 2:** Distribution of genotypes and alleles for *FAHH* c.385C>A SNP in anorexia nervosa and control groups

		Genotype, *n* (frequency)				Allele, *n* (frequency)			
				
Groups	*n*^1^	AA	CA	CC	*P*^2^	A	C	*P*^2^	OR (95% CI) for minor allele
All AN	762	22 (0.029)	190 (0.249)	550 (0.722)	0.039	234 (0.154)	1290 (0.846)	0.028	0.799 (0.653–0.976)
RAN	375	8 (0.021)	89 (0.237)	278 (0.741)	0.026	105 (0.140)	645 (0.860)	0.0094	0.717 (0.557–0.922)
PAN, BAN, ANBN	387	14 (0.036)	101 (0.261)	272 (0.703)	0.23	129 (0.167)	645 (0.833)	0.29	0.880 (0.694–1.117)
Control	605	18 (0.030)	188 (0.311)	399 (0.660)		224 (0.185)	986 (0.815)		

RAN, lifetime anorexia nervosa restricting subtype; PAN, lifetime anorexia nervosa purging subtype; BAN, lifetime anorexia nervosa with binge eating; ANBN, lifetime anorexia nervosa and bulimia nervosa; All AN, RAN, PAN, BAN, and ANBN; OR, odds ratio; 95% CI, confidence interval.

1 Number of samples genotyped successfully.

2 Chi-square test.

We estimated that the detection power of our sample size for allele-wise association analysis between all AN subjects and the control group was 58%, when the effect size of the odds ratio = 1.25 (1/0.799), 385C allele frequency in controls = 0.815, and *α* = 0.05.

The lifetime minimum BMIs of the AN patients were 13.0 ± 2.52, 12.7 ± 1.94, and 12.9 ± 2.12 kg/m^2^ for the AA (*n* = 21), CA (*n* = 171), and CC (*n* = 485) genotypes, respectively. There was no significant difference in minimum BMIs between the 385A allele carriers (AA and CA, 12.7 ± 2.01 kg/m^2^) and noncarriers (CC, 12.9 ± 2.12 kg/m^2^) (one-way ANOVA, *P* > 0.05).

## Discussion

The present findings indicated that the *FAAH* c.385C>A SNP was moderately, but significantly, associated with lifetime AN. The minor 385A allele was less frequent in the AN patients, especially in lifetime restricting AN patients who had not engaged in binge eating and purging in their history.

The c.385C>A SNP is a nonsynonymous and functional polymorphism that converts a conserved proline residue of FAAH to threonine (p.Pro129Thr) (Sipe et al. 2002). The *FAAH* c.385C>A SNP missense polymorphism codes for a functionally deficient protein, and subjects with this polymorphism have ∼50% FAAH protein expression and enzymatic activity when compared with the wild-type enzyme (Chiang et al. 2004). Thus, this reduction in FAAH expression and activity possibly increases AEA levels in the brain (Cravatt et al. 2001), enhances appetite and food intake through the stimulation of CB1R (Berry and Mechoulam 2002), and counteracts starving behavior (Hao et al. 2000), which may be protective against the development or maintenance of underweight AN.

When the AN group was subdivided into lifetime restricting AN and binge eating/purging AN patients, only the restricting AN group exhibited a significant association with the *FAAH* c.385C>A SNP. This may suggest that the variant is related to subtype change in AN, because those carrying the 385A allele seem to be more prone to overweight (Sipe et al. 2005; Monteleone et al. 2008). Nevertheless, the frequency of the 385A allele in the binge eating/purging AN group was still lower than in the control group. Therefore, the absence of a significant difference between the binge eating/purging AN patients and the control group may be simply due to the reduction in statistical power by subgrouping.

Monteleone et al. (2009) first reported the associations of the c.385C>A SNP of *FAAH* with AN and BN. They observed a higher frequency of the minor A allele in their AN and BN groups, which is opposite to the lower frequency of the A allele observed in our AN group. The reason for these conflicting results might be the relatively modest sample size (134 AN, 180 BN, and 148 control participants) and tendency for the deviation of genotype distributions from HWE in the previous study (Monteleone et al. 2009). Conversely, Müller et al. (2008) did not find any association with the same SNP of *FAAH* with AN in their TDT study with 91 trios and case–control study with 113 AN patients and 178 controls. Again, their sample size was modest and its statistical power should be limited. Recently, two GWAS did not report an association of the FAAH c.385C>A SNP with AN (Wang et al. 2011; Boraska et al. in press). However, no SNPs reached genome-wide significance in these two GWAS, indicating that they were underpowered.

Although the endocannabinoid system plays an important role in the regulation of feeding behavior and the rewarding properties of food (Pagotto et al. 2006), the genetic variation in *FAAH* may affect susceptibility to AN through other mechanisms. The low-dose administration of anandamide not only increases food intake but also improves cognitive function and reverses neurotransmitter changes caused by diet restriction (Hao et al. 2000). The disruption of FAAH activity has anxiolytic effects that are mediated by CB1R stimulation (Kathuria et al. 2003). A high prevalence of anxiety disorders is observed in AN, which commonly precede the onset of the disorder (Strober 2004). Carrying the 385A allele may decrease susceptibility to AN by reducing anxiety.

Genetic association studies concerning eating disorders have been performed on other genes belonging to the endocannabinoid system and have yielded conflicting results. Siegfried et al. (2004) tested the association of the (AAT)_n_ repeat in the 3′-flanking region of *CNR1* in their family-based study and suggested that restricting AN and binging/purging AN may be associated with different alleles of *CNR1*. However, Müller et al. (2008) did not find evidence for an association of the (AAT)_n_ repeat or for several SNPs in *CNR1*, *FAAH*, *NAAA*, and *MGLL* with AN in a TDT and case–control study. Monteleone et al. (2009) showed that the *CNR1* c.1359G>A SNP, in addition to the *FAAH* c.385C>A SNP, was significantly associated with AN and BN, and that there is a synergistic effect of both SNPs on AN. Nevertheless, the *FAAH* c.385C>A SNP is the only variant with any evidence of functional significance among the *FAAH*, *CNR1*, *NAAA*, and *MGLL* variants reported above. In addition, the minor allele frequency of the *FAAH* c.385C>A SNP only provides moderate power to detect a possible difference in allele frequency between groups, even with the sample size used in this study.

Although the sample size in this study is larger than in any other prior study of the genetic association of the *FAAH* c.385C>A SNP with eating disorders, the results should be confirmed by independent studies. A case–control study is prone to the influence of population stratification, however, all of our participants were Japanese females. In addition, the frequency of the 385A allele in our control subjects (0.168) was similar to that reported in a previous study by Iwasakia et al. (2007) (0.171) in 799 Japanese healthy controls. Therefore, the association between AN and the *FAAH* c.385C>A SNP in our sample is unlikely to be due to the deviation of allele frequencies in the control population.

In conclusion, the current results suggest that the minor 385A allele of the *FAAH* gene is less frequent in AN patients and having this allele is possibly protective against AN, especially restricting AN. Our findings also support the hypothesis that the endocannabinoid system is involved in the etiology of AN and can be a promising target of further genetic studies as well as potential therapeutic manipulation (Marco et al. 2012).
